# From invaders to residents: The golden jackal (*Canis aureus*) expansion in Hungary since the mid-1990s

**DOI:** 10.1371/journal.pone.0306489

**Published:** 2024-07-11

**Authors:** Hanna Bijl, Gergely Schally, Mihály Márton, Miklós Heltai, Sándor Csányi

**Affiliations:** Department of Wildlife Biology and Management, Institute for Wildlife Management and Nature Conservation, Hungarian University of Agriculture and Life Sciences, P áter Károly, Gödöllő, Hungary; University of Agricultural Sciences and Veterinary Medicine Cluj-Napoca, Life Science Institute, ROMANIA

## Abstract

The golden jackal has rapidly expanded across Europe in recent decades and was one of the first to reappear in Hungary. Using hunting bag data from the National Game Management Database from 1995 to 2021, we examined the spatial expansion of the species and its population dynamics. Our findings reveal an exponential increase in the golden jackal population since the mid-1990s, with an average annual growth rate of 40% in the hunting bag and an occupancy of 86% of the country. The average speed of the range expansion was 536.9 km^2^ year^-1^ until 2007 and increased to an average of 5,289.1 km^2^ year^-1^ during the second part of the expansion process. The density of shot golden jackals also shows an increasing trend with a substantial presence of outliers, indicating that nearly 10% of the hunting bag demonstrates exceptionally high numbers, particularly in southwestern Hungary. The spread originated in the southwest and extended towards the northeast and northwest. However, the increase of the hunting bag slowed down and the expansion rate has decreased in the previous two years, leading us to anticipate that the golden jackal population will stabilize soon, as the expansion has reached its maximum in Hungary. This indicates that from the initial settlement to the stabilization of the population, the time span can be two to three decades.

## Introduction

The golden jackal (*Canis aureus*) is a native meso-carnivore in the Northern Hemisphere that has shown a rapid expansion in Europe in the last few decades [1–3]. Initially, jackals were restricted to only minor patches of the Mediterranean and Black Sea coastal areas [4, 5]. However, the core populations and historical distribution remain understudied [5]. From the 1970s, the expansion occurred quickly towards south-eastern and central Europe, where reproducing populations have been established since [6–11]. The expansion from the Balkan Peninsula is still in motion towards northernmost and westernmost Europe where vagrant individuals have been observed in previous years [12, 13] or reproduction has been confirmed [14–16].

Hungary was one of the first countries where the species reappeared in Europe in the 1980s after it had been mostly absent since the 19^th^ century [17]. Moreover, the species was considered extinct in 1989 as there were no reproducing individuals between the 1940s and 1990s [18], after the last known and officially reported individual was shot in 1942 [19, 20]. However, the species’ return has accelerated since the early 1990s and became a game species in 1994 with a year-round hunting season until 1999 and again from 2012 [20, 21]. Between 1999 and 2012, the species could be hunted from the first of July until the end of February.

The study on the spread of the species in Hungary has been limited to the initial phase of its expansion. Previous research by Szabó et al. (2009) [20] revealed an exponential growth of the golden jackal population in Hungary from 1997 to 2006 with a hunting bag of 11 to 163 individuals. Additionally, stable populations were observed in the country’s southwestern region [20]. Building on these findings, we hypothesized a further exponential increase of the population size and an accelerating increase of the range from the southwest area (initial population) until the species has covered nearly all country. Subsequently, after reaching a maximum, we anticipated a decline in the population growth rate for the previous years.

Thus, this study aims to broaden the existing knowledge about the population dynamics of the golden jackal over the past three decades based on hunting bag data. In this study, we determined (1) the changes in annual increase in the population size/hunting bag of the golden jackal in Hungary between 1995 and 2021 (numerical dynamics); (2) the changes in the yearly increase in the range of the species (spatial expansion) in the country during this period, (3) changing of the population density according to the shot individuals (density growth rate), and (4) changes in the increasing of the spatial rate (aerial speed of expansion) during this period. Moreover, hunting bags were used as the most comprehensive indicators of the population changes of the golden jackal populations living in Hungary.

## Materials and methods

### Data collection

Official hunting bag data was used from 1995 until 2021 from Hungary’s National Game Management Database (NGMD), obtained from Game Management Units (GMUs). The animals were legally hunted according to the Hungarian Game Management and Hunting Law (the Act on Game Conservation, Management and Hunting (Act LV, 1996) [22]). GMUs in Hungary are hunting areas with a minimum of 3,000 ha and have a distance between the borderlines of at least 3,000 m [23]. Every GMU is obligated to report the hunting bag and population numbers, and this data is collected by the NGMD every year at the end of the hunting season (late February). Given that the population numbers are rather guesstimates, our analysis solely relied upon hunting bag data, assuming that these data serve as a consistent proxy for the population size [24, 25]. Furthermore, GMUs change in size and numbers over time. Depending on changes in legislation and/or landownership, borders might change regularly or unexpectedly. Therefore, to prevent bias in space and to overcome the problems of changing GMUs, the Universal Transverse Mercator (UTM) grid system was used with a grid resolution of 10 × 10 km [26]. The borders of the GMUs in Hungary and the UTM grid system were intersected, and the ratios of the areas belonging to different UTM grid cells compared to the total size of the GMUs were calculated. Hunting bag data was then multiplied by this ratio for each GMU and these values were summarized and rounded for each UTM grid cell.

### Data processing

The data was analyzed both temporally and spatially. The temporal analysis shows the changes in hunting bag, distribution area, and annual increase in the distribution area of the golden jackal in Hungary from 1995 to 2021. To model the phase transformations over time, a best-fitted curve was plotted using the Avrami equation [27, 28]:

$$f(x)=A∙(1-EXP(-B∙t^{n}))$$

with constants A, B, and n. The sum of the squared residual (SUM = (real y value–y value of the equation)2) (SSR) was calculated, and then Solver in Microsoft Excel was used to change the values for A, B, and n (*t* is equal to the x value) to minimize the SSR. Moreover, we also looked at the changes in the density of shot golden jackals per km^2^ (hunting bag/UTM grid cell). Additionally, we made a frequency distribution with the density of shot golden jackals (per km^2^) based on 17 data bins corresponding to intervals of “0–0.1,” “0.1–0.2,” “0.2–0.3,” and so on until the maximum density of 1.8 was reached. This way, the distribution of occupied UTM grid cells from 1995 to 2021 was the best visible. As the density of shot golden jackals per km^2^ showed many outliers yearly, we identified and highlighted these outliers spatially based on the IQR criterion.

For the spatial analysis, two non-linear regression models (one polynomial and one power) were fitted to describe the relationship between (1) the number of occupied UTM grid cells (x) and the annual increase in the number of occupied grid cells by the golden jackal (y), and (2) the surface area occupied (x) and the annual increase in surface area (%) (y). The normality of the models’ residuals was tested using the Shapiro-Wilk test and the dependence between the independent variables (number of occupied UTM grid cells and occupied surface area) and the residuals were tested graphically. The explained variance rates (R^2^) and the parameter estimations were tested by t-tests. The analyses were performed in RStudio software (v.4.02.32.) [29].

## Results

### Temporal analysis

**Fig 1:**
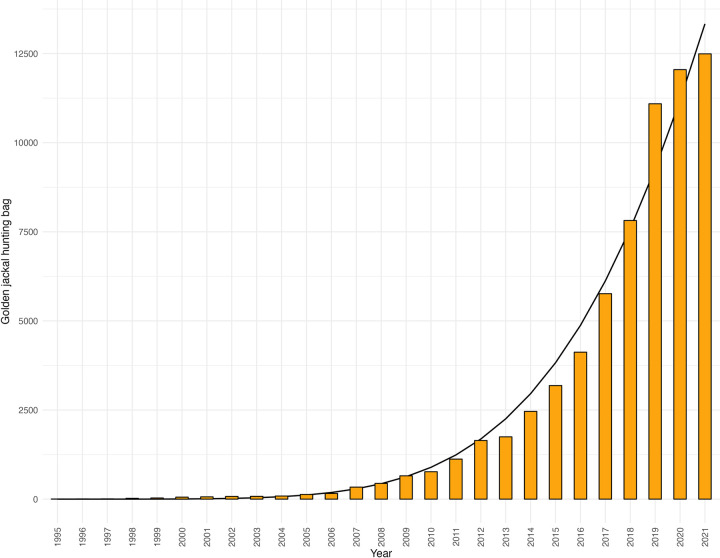
Golden jackal hunting bag in Hungary from 1995 to 2021 with the best-fitted curve.

Between 1995 and 2021, the hunting bag of the golden jackal showed a robust, strong increasing trend; exponential growth until 2019 and a deceleration in the most recent two years (Fig 1). The hunting bag has an average increase of 40.03% annually (min = 2.63%; max = 175.00%) and average λ of 1.40 (λ min = 1.03; λ max = 2.75). By fitting the best-fitted curve (Avrami equation) of 44,341(1–exp(–5.13E–09 ∙ *t*^5.48^)), it is anticipated that the population growth will continue to decline in the near future, leading to a stabilization of the population.

**Fig 2:**
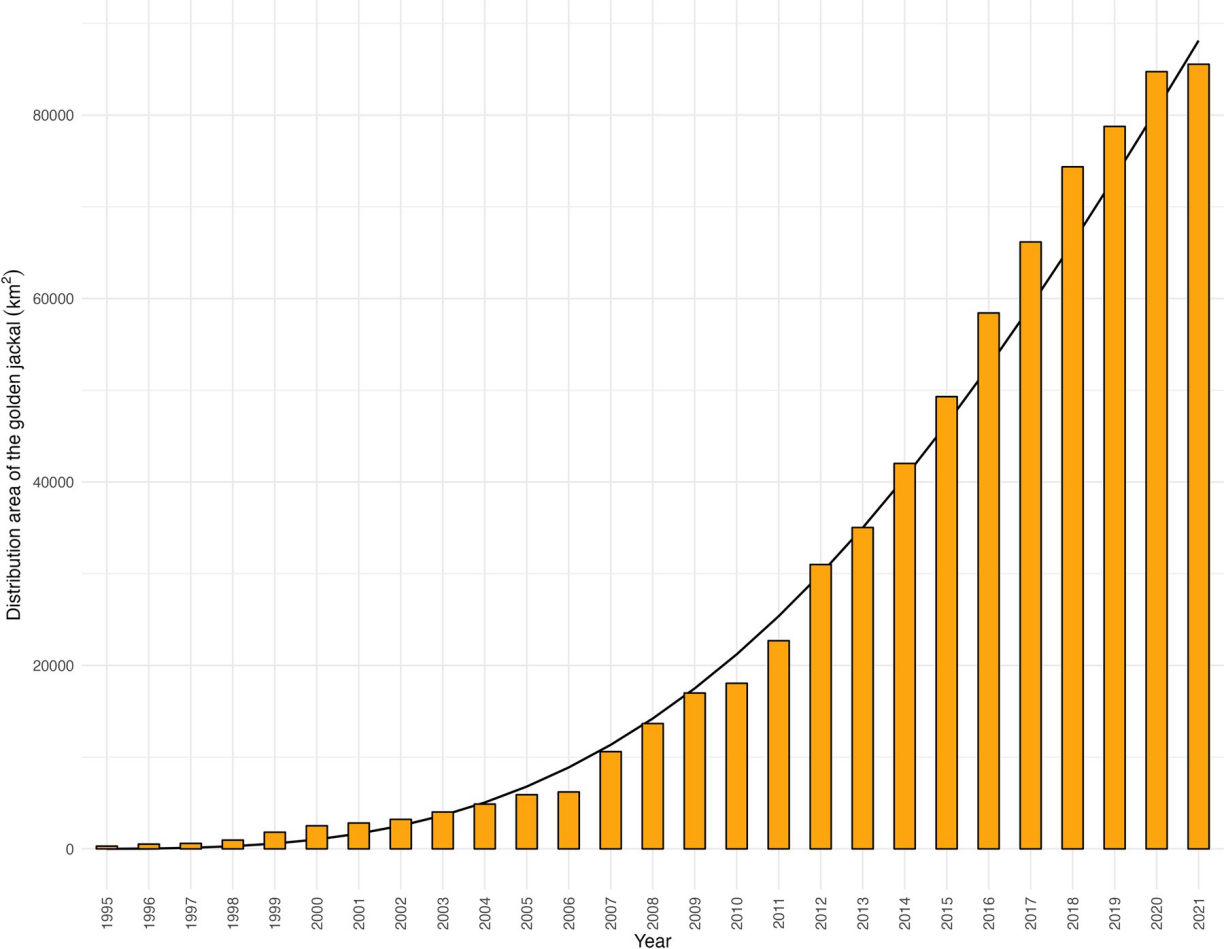
Distribution area of the golden jackal in Hungary from 1995 to 2021 with the best-fitted curve.

During the study period, the area occupied by the golden jackal increased by over 85,000 km^2^ (Fig 2). Similarly to the hunting bag, a slight deceleration can be observed in the last two years. Currently, the golden jackal occupies 86% of the country, compared to 0.32% in 1995. The best-fitted curve of 200,905(1–exp(–1.84E-05 ∙ *t*^3.14^)) shows that a logistic curve might develop in the foreseeable future, given the current saturation of the country’s habitat by the golden jackal.

**Fig 3:**
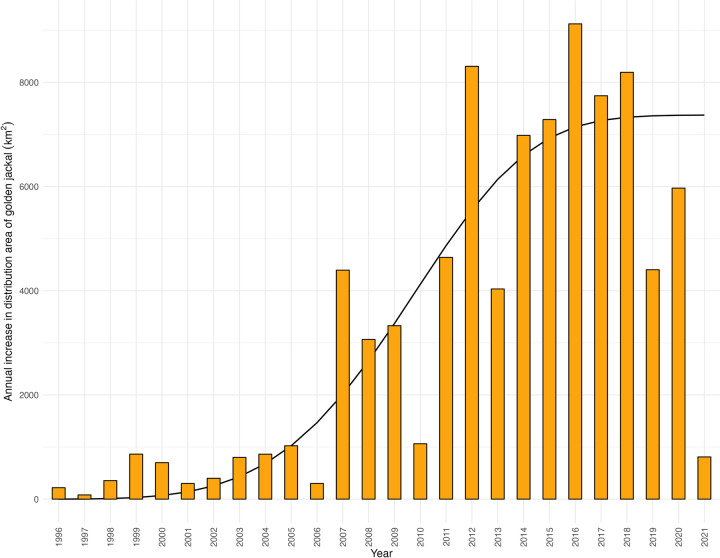
Annual increase in the distribution area of the golden jackal in Hungary from 1996 to 2021 with the best-fitted curve.

Regarding the annual increase in the distribution area, a difference can be noticed between the first half of the study period (1995–2006) and the second half (2007–2021), suggesting a two-phased expansion process (Fig 3). The average annual increase in the first period was 536.9 km^2^ year^-1^ (min = 80.7; max = 1,024.0 km^2^ year^-1^). The average annual increase in the second period was 5,289.1 km^2^ year^-1^ (min = 810.0; max = 9,122.2 km^2^ year^-1^). The best-fitted curve of 7,372(1–exp(–2.77E–06 ∙ *t*^4.54^)) shows a visible logistic curve. However, as the population expansion has slowed down in the country, a further decrease may be observed soon, leading to a Gaussian curve.

**Fig 4:**
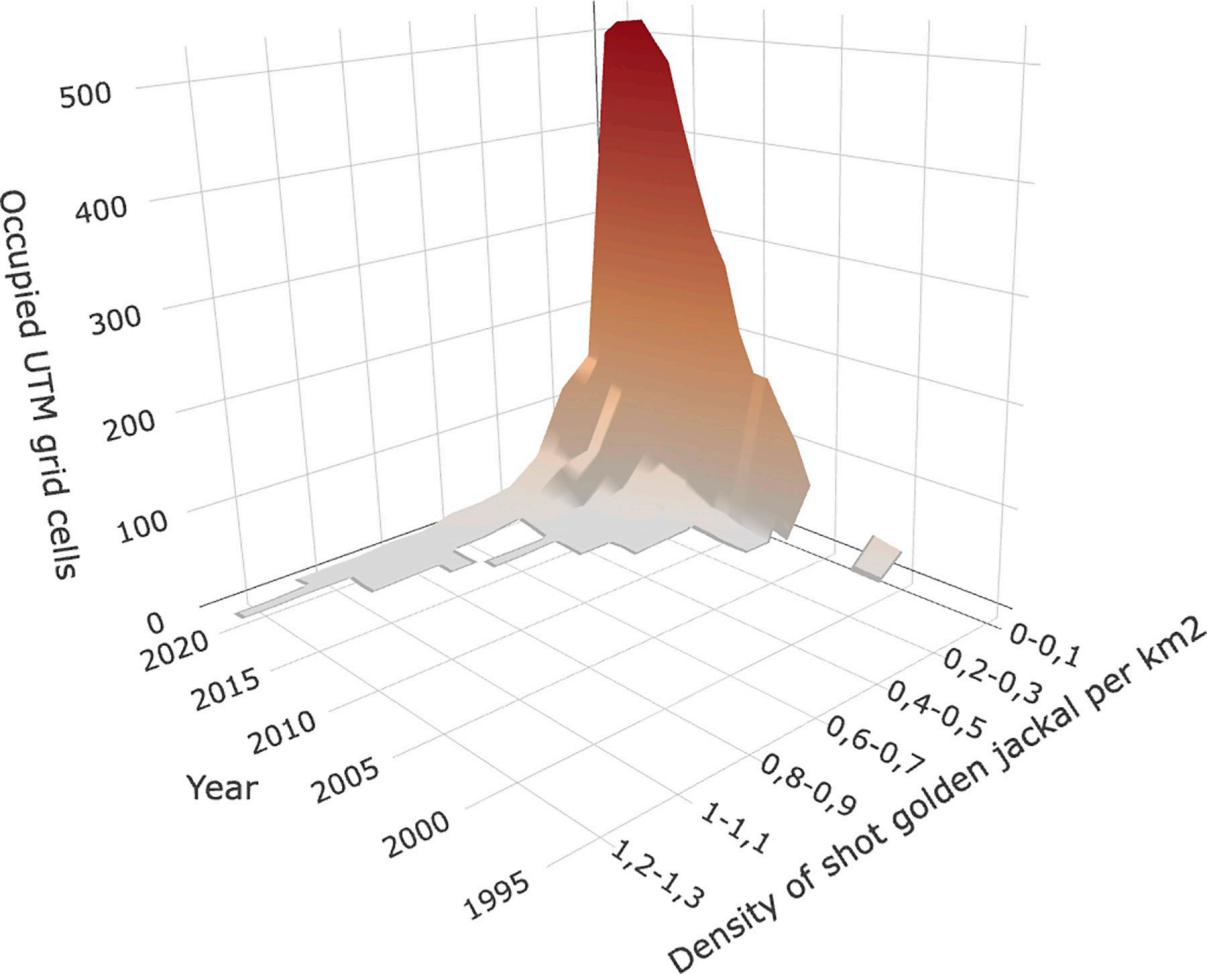
Frequency distribution of occupied UTM grid cells according to 17 density categories of shot golden jackals per km^2^ in Hungary from 1995 to 2021.

Concerning the frequency distribution of occupied UTM grid cells, the density of shot golden jackals was divided into 17 data bins (Fig 4). Most UTM grid cells are in the lowest data bin (0–0.1) and only a few UTM grid cells show a higher density, although these have increased in the past few years too. Only from 2019 have golden jackals occupied UTM grid cells in every category.

**Fig 5:**
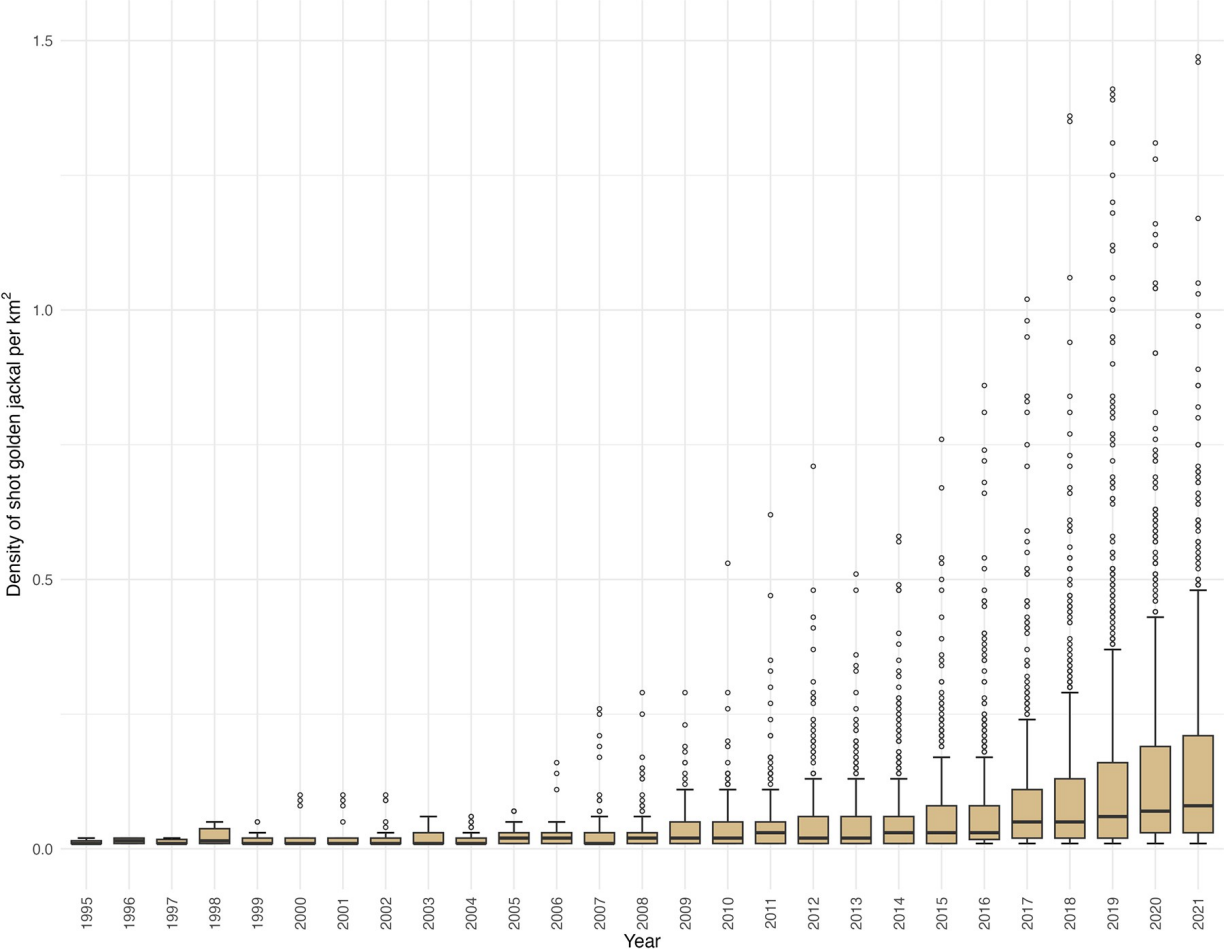
Density of shot golden jackal per km^2^ in Hungary from 1995 to 2021.

The density of shot golden jackals per km^2^ exhibited a relatively slow increase since 1995, even after removing zero values (Fig 5). In 2021, the average density of shot golden jackals/km^2^ was 0.15 (min = 0.01; max = 1.47). On the other hand, instances of outliers began to emerge in 1999 and have shown a consistent upward trend, except for 2003, when no outliers were identified. On average, 9.31% of the hunting bag comprised outliers (min = 4.76%; max = 21.05%), indicating that nearly 10% of the hunting bag demonstrated exceptionally high numbers, especially in southwestern Hungary (Fig 8). Notably, a similar pattern of decreasing number of outliers has been observed in the past few years, aligning with the trends seen in the previous graphs.

### Spatial analysis

**Fig 6:**
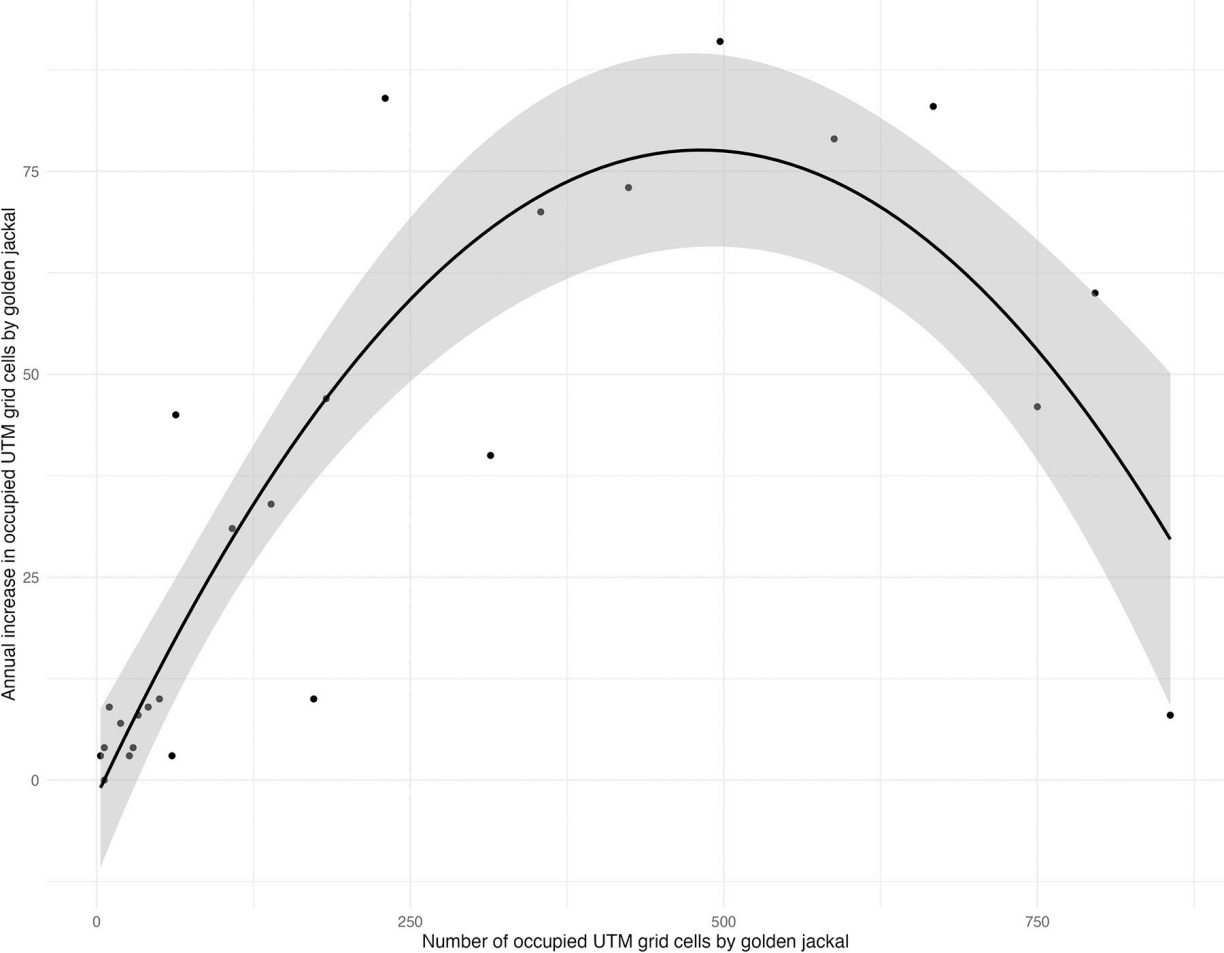
Regression analysis of the number of occupied UTM grid cells by golden jackals and the annual increase in occupied UTM grid cells by golden jackal in Hungary.

The relationship between the number of occupied UTM grid cells and the annual increase in occupied UTM grid cells shows that the golden jackal expansion has passed its maximum as the annual increase of occupied cells has decreased when more than 500 cells (equivalent to 50,000 km^2^) were occupied by the golden jackal (Fig 6). The best-fitted function is y = 0.0003x^2^ + 0.3302x – 1.9353 with explained variance rate R^2^ = 0.788; *t*(24) = 9.44; p < 0.001 for the polynomial regression.

**Fig 7:**
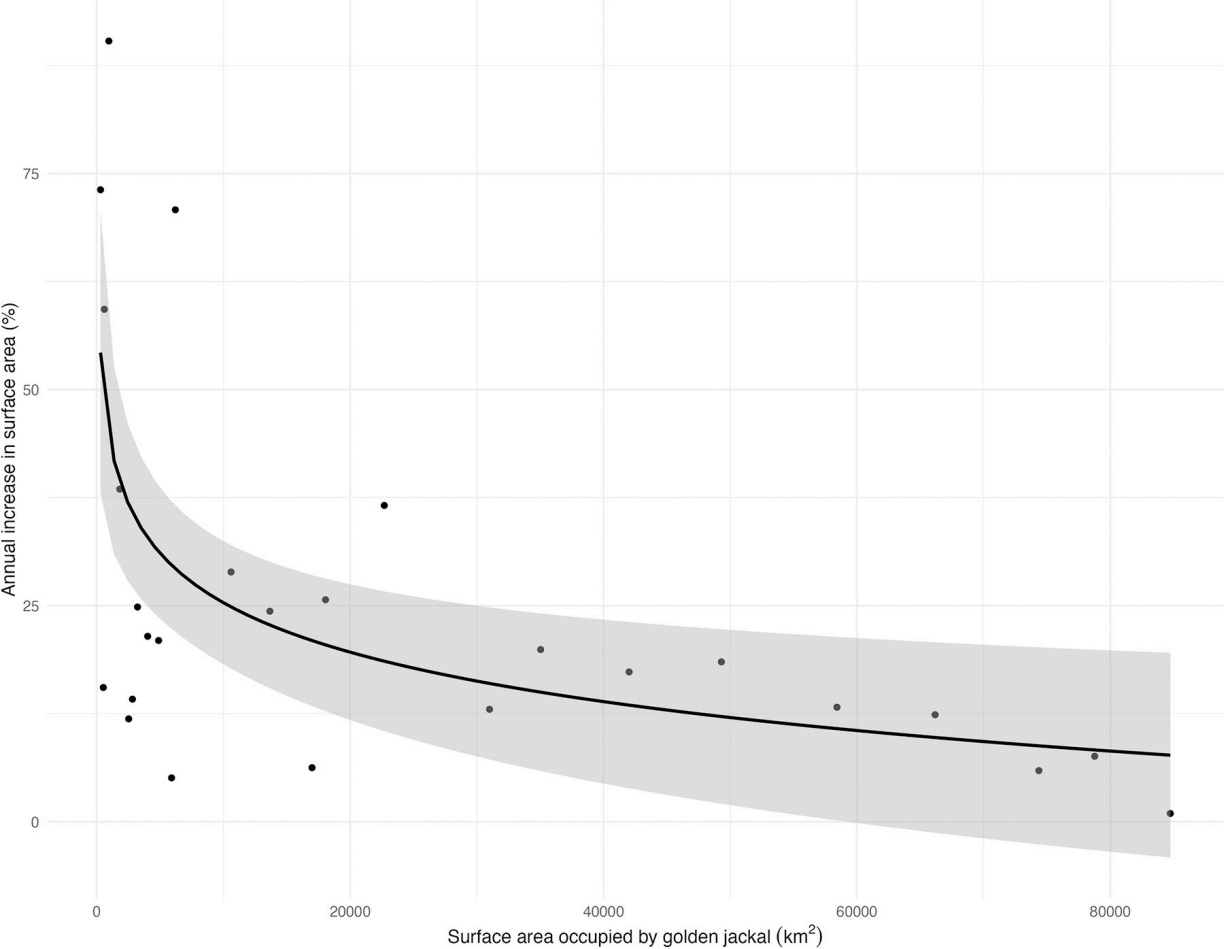
Regression analysis of the surface area occupied by golden jackal and the annual increase in surface area in Hungary.

The relationship between the area occupied by the golden jackal and the annual area increase shows that the rate of expansion was in the range of 15–25% per year, except for a few initial years (Fig 7). Moreover, this model shows that the annual area increase was the highest when only a few areas were occupied; however, this quickly decreased when more spaces were occupied and tended to keep a decreasing trend the more elevated the occupancy gets. The best-fitted function is y = 377.09x^-0,334^ with explained variance rate R^2^ = 0.343; *t*(24) = 3.79; p < 0.001 for the power regression.

### Movement of range expansion

**Fig 8:**
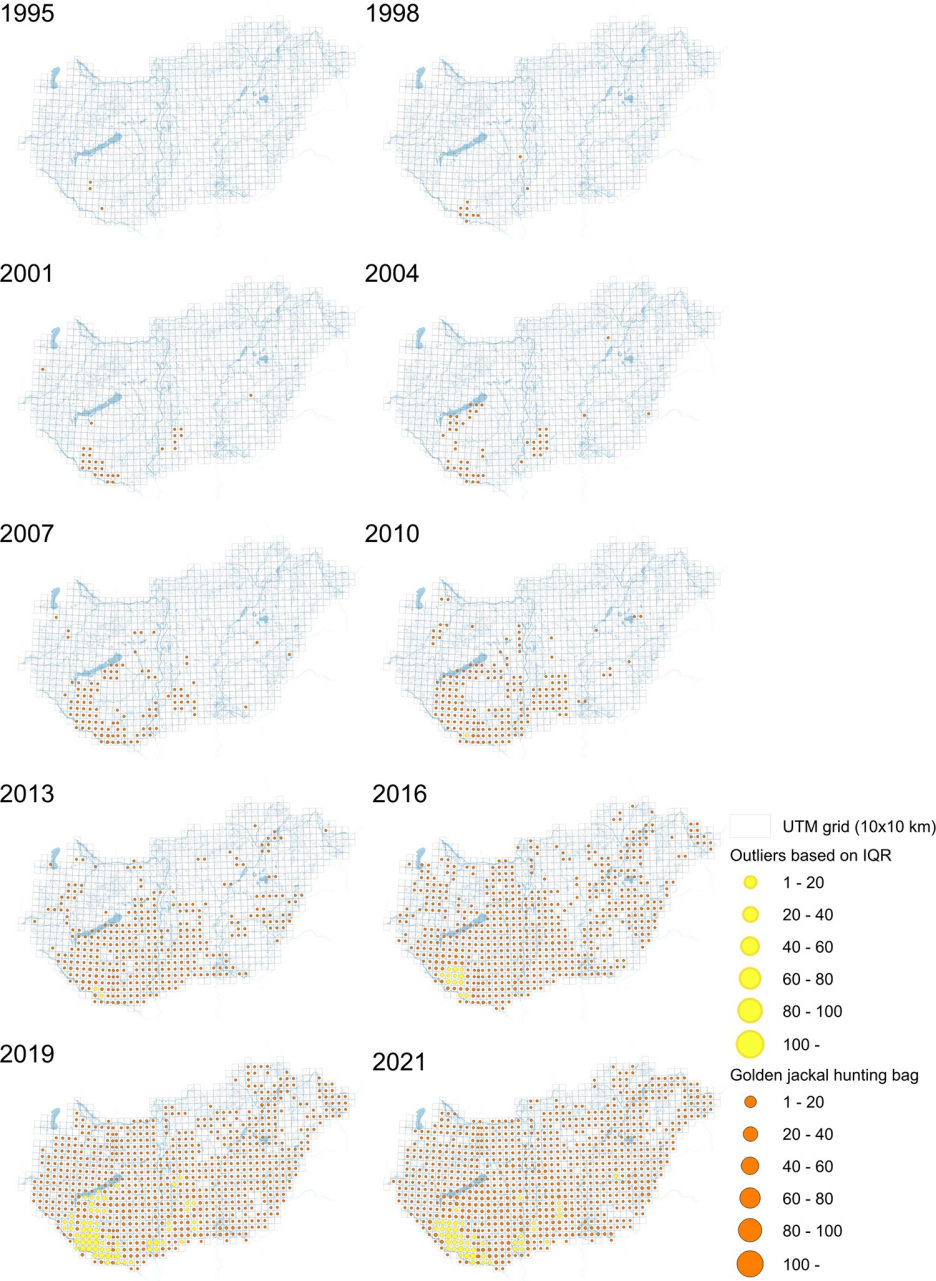
Distribution maps of the golden jackal hunting bag in Hungary from 1995 to 2021 with three-year intervals and outliers based on IQR highlighted in yellow. Sources: Horváth et al. (2008) [26] and openstreetmap.org [30].

The range expansion of the golden jackal in Hungary started in the southwestern region, specifically above the Drava River (Fig 8). According to the hunting bag, solitary individuals or small groups briefly appeared in 1997 in central Hungary but subsequently disappeared, only to re-emerge in 2000, occupying a more significant number of neighboring grid cells. However, it could be that the species was already present, but not taken, resulting in “empty” grid cells. Concurrently, single-occupied grid cells emerged in the western and eastern parts of the country. Notably, the hunting bag in the southwestern region, encompassing Somogy and Baranya counties, expanded into adjacent grid cells extending towards Lake Balaton. Consequently, the hunting bag in these two counties has increased to 20–40 individuals per grid cell (100 km^2^) by 2007. These populations demonstrated rapid growth, meanwhile new populations were established in the northeastern and northwestern parts of the country. These newly settled populations further expanded, occupying additional grid cells in the central region. At the same time, the hunting bag in the southern region experienced an increase to over 100 shot individuals per grid cell. The golden jackal has successfully colonized the entire country, except for the northernmost and north-western areas.

When considering the (highlighted) outliers, it is clear that the largest number of golden jackals are shot in the southwestern and, to a lesser extent, in the central part of the country. These are also the areas where the golden jackals initially appeared, and can be regarded as core areas. Moreover, the number of outliers reduced in the previous two years, with a peak in 2019 (*N* = 71, 8.92%; *N* = 55, 6.43% in 2020; *N* = 45, 5.21% in 2021).

## Discussion

### Expansion of the golden jackal in Hungary within European context

The golden jackal in Hungary has exhibited rapid exponential growth since its reappearance in the early 1990s, with recent indications of a slowdown in population expansion during the last two years. The hunting bag data shows an annual increase of 40%. Although hunting pressure depends on various factors, such as location, strategy, and season [31], the observed increase in the hunting bag must reasonably reflect the expanding population size.

Once established within the country, the species could show accelerated development of reproductive capacity and territorial groups, similar to other countries within the core range of the jackal population in the Balkan Peninsula. For example, in Greece, the population grew 327% in 16 years [32], while Bulgaria experienced a 33-fold increase in population size between the 1960s and 1980s [33]. Similarly, Bosnia and Herzegovina observed an average annual increase of 35% in the hunting bag from 2000 to 2016 [8]. Also Romania, Hungary’s neighboring country, recorded a 6.7-fold exponential growth from 1,871 specimens to 12,206 between 2006 and 2017 [34]. However, in European countries outside of the core range, the golden jackal exhibits a prolonged lag phase with a gradual population build-up. For instance, in Slovenia, it took 30 years for the species to grow exponentially after regular presence in the country [35]. Similarly in Italy, the colonization is limited and slow [36], and in Germany territorial groups were formed 23 years after the first record in 1997 [37, 38].

The high velocity (speed) of the range expansion in Hungary depicts an expansion process similar to that of invasive species [20, 39]. No such velocity has been reported in native (canid) species before. After all, native species can show traits similar to invasive species, such as rapid spread, occupancy of novel areas, and ecosystem dominance, and can be defined as “native invasive species” or “native invaders” [40]. However, most studies documenting a species’ spread are traditionally focused on exotic organisms becoming invasive [41].

Furthermore, Bulgaria has been identified as having the largest jackal population, estimated at 39,343 individuals in 2011 [42]. However, considering the hunting bag of over 12,000 individuals in 2021, the actual golden jackal population in Hungary could reach 60,000 individuals, assuming that one-fifth of the population is removed through hunting. Thus, making Hungary a significant contributor, if not the primary host, to the European jackal population.

### Drivers for expansion

These differences in population growth between countries could be attributed to the availability of anthropogenic food sources [5] and the associated waste management regimes [35, 43]. This is because organic waste is an important food source for golden jackals [44]. Besides food availability, changes in agricultural intensity and climatic conditions could have been other expansion drivers [5, 45]. In particular, the lack of marsh habitat and long-lasting snow cover can be determining factors [46, 47]. Also, mountainous areas can limit or at least slow down the spreading, particularly altitudes of up to 1,000 m a.s.l. have shown only a few golden jackals [6], and altitudes of over 1,200 m a.s.l. have shown no detection of golden jackals [48].

Furthermore, it is often suggested that the absence of the grey wolf (*Canis lupus*) also facilitated the expansion [2, 49] due to the lack of interference competition. Although these are complex interactions and depend on numerous factors, it can be acknowledged that large carnivores can coexist alongside mesocarnivores where sufficient food resources and suitable hiding places are available [50]. Therefore, the presence of the grey wolf can be considered a limiting factor for the expansion of golden jackals to some extent; but it does not entirely preclude the occurrence of jackals in areas inhabited by wolves. This has also been evident in Hungary, where the presence of the golden jackal has been confirmed within wolf territories. However, here the golden jackals are (still) only in small numbers and mainly at the edges of wolf territories [51].

Moreover, the red fox (*Vulpes vulpes*) is the primary competitor of the golden jackal in the canid guild in Hungary, yet it seems not to hamper the expansion, even though the red fox hunting bag has increased almost threefold during the same period [52]. Both species are sympatric canids; the golden jackal may be more dominant due to a narrower trophic niche breadth, more successful offspring rearing, engagement in group hunting, and overall larger body size [53, 54]. This is supported by Farkas et al. (2017) [55], where the body weight of juvenile rex foxes is affected by the nutritional niche overlap between the two species.

The expansion of the golden jackal in Hungary is likely attributed to changes in land use, characterized by the reduction in agricultural areas and an increase in forest cover since the 1950s [56]. Consequently, similar to most European countries, the abundance and harvest of all common deer species have increased noticeably since the 1980s [57–59]. These cervids can provide a natural prey basis for golden jackals during the rearing period of the offspring, when the fawns are still hiding [60, 61]. This rise in cervid game populations has also resulted in a substantial availability of leftover viscera and carrion from deer hunters, a considerable component of Hungary’s golden jackal’s diet [43]. This provides the golden jackals with a supplementary protein source for seven to nine months. Additionally, the main diet component of the species consists of small mammals (i.e. voles, (dor)mice, coypu, rodents, moles, shrews, (musk)rats) [62]. Hungary’s increased suitable forest habitat may have contributed to the concurrent increase in small mammal populations (e.g., bank voles (*Myodes glareolus*) [63]). However, the abovementioned drivers for the expansion are only speculations, and further investigation into the influence of environmental factors (in Hungary) is needed.

### Harvest mortality compensation

When analyzing the density of shot golden jackals in Hungary, a prominent concentration becomes evident in the country’s southwestern part, specifically in Somogy and Baranya counties. Over time, this hot spot has gradually expanded and slightly moved towards the northeast. This observation suggests that non-reproducing females may be participating in reproduction as a compensation for the high mortality rate, as typically only alpha females reproduce within the family group [54]. This is supported by Pecorella et al. (2023) [64], where subordinate females have potentially participated in reproduction within the same unit. Moreover, neighboring individuals from adjacent areas could also occupy vacant territories, thereby contributing to maintaining the overall high population size in the region [65]. This has been documented in Hungary, where golden jackals can form a new pair bond after the alpha female deceased [66]. Furthermore, the average population density in the Balkan Peninsula varied between 0.6 and 1.1 territorial groups per 10 km^2^, implying that within a 100 km^2^ grid cell, there could be six to 11 groups and potentially even up to 48 groups [45]. Correspondingly, Hungary’s average home range of the golden jackal is 11.2 km^2^ [67]. These would support our findings of many outlier cells, as many territorial groups could readily occupy empty areas where individuals have been hunted.

### Management implications

The golden jackal is a highly adaptable species that is not quickly limited by local resources. Consequently, the species has made a penetrating return in the European fauna and even appears in areas where it has never occurred before [e.g. 15, 16]. Accordingly, the management implications of the species depend on its status (i.e. game, protected, not classed), which seems to be strongly divided between European countries [68]. The presence of golden jackals could result in conflicts, such as predation on livestock [69] or endangered and game species, thus posing a higher risk of spreading diseases [10], although these incidents remain minimal. Nonetheless, in areas with high golden jackal concentration, the main focus for management should be mitigating nuisance or damage from predation. Preferably, density-dependent methods like trapping should be employed, as hunting alone has proven insufficient in Hungary for effectively reducing population size. Moreover, it shows that from the initial settlement to the stabilization, the time span can be two to three decades. Predictions based on limited information, the lack of data, or misinterpretation of evidence may hinder the appropriate management actions.

Based on our findings, we anticipate that the golden jackal population will stabilize soon, as the expansion has reached its maximum in Hungary, assuming the environmental conditions remain relatively stable. Simultaneously, the species is continuing its expansion north-northwest to Slovakia and Austria. It is advised to monitor the species to understand its dispersal behavior better to lessen the population explosion that is likely to occur in western and northern European countries.
